# Evaluation of the quality of life of children and adolescents with type 1 diabetes mellitus before and after an intervention with a remote multiprofessional educational program

**DOI:** 10.1016/j.jped.2026.101537

**Published:** 2026-04-03

**Authors:** Camilla Kapp Fritz, Andreia Araújo Porchat de Leão, Fernanda Volpato França Sbalqueiro, Cesar Augusto Taconeli, Claudia Choma Bettega Almeida, Marcia Regina Messaggi Gomes Dias, Suzana Nesi França

**Affiliations:** a Universidade Federal do Paraná, Graduate Program in Child and Adolescent Health, Curitiba, PR, Brazil; b Universidade Federal do Paraná, Hospital de Clínicas, Curitiba, PR, Brazil; c Universidade Federal do Paraná, Department of Statistics, Curitiba, PR, Brazil; d Universidade Federal do Paraná, Department of Nutrition, Curitiba, PR, Brazil; e Universidade Federal do Paraná, Department of Pediatrics, Curitiba, PR, Brazil

**Keywords:** Diabetes mellitus type 1, Telemedicine, Quality of life, Technology

## Abstract

**Objective:**

This study aims to evaluate the quality of life (QoL) and glycemic control of children and adolescents living with type 1 diabetes mellitus (T1D) before and after an intervention with a multidisciplinary educational program.

**Method:**

In this prospective interventional study, 47 participants were divided into an intervention group (IG), which underwent a six-month in-person and online multiprofessional education program, and a control group (CG), which received standard in-person follow-up. After three months, all participants in this study had a continuous interstitial glucose monitor (CGM). QoL was assessed by the Pediatric QoL Questionnaire PedsQL 3.0 – Diabetes Mellitus Module. Scores were separated into tertiles.

**Results:**

Both groups had homogeneous general characteristics and mean HbA1c above the recommended levels. The QoL level in the IG and its guardians at T180 showed no differences but girls showed lower QoL scores at T0. Participants in the CG with intermediate perceptions showed a reduction in their QoL in the category “barriers to treatment” in relation to IG. The IG showed a significant increase in adherence to the carbohydrate counting method (CHCM). QoL, according to the parents’/guardians’ perception, was lower than that of their children.

**Conclusions:**

The intervention failed to significantly affect glycemic control either in HbA1c or in time in the therapeutic range. The intervention and CGM failed to improve glycemic control and QoL in individuals living with T1D and suboptimal glycemic control. However, it significantly increased adherence to CHCM.

## Introduction

Brazil ranks third in the world for individuals under 20 years of age with type 1 diabetes mellitus (T1D), representing a serious public health proble [[1], [2]].

T1D constitutes a chronic disease that requires arduous daily management, including glycemic self-monitoring, multiple insulin applications, adequate nutrition, a carbohydrate counting method (CHCM), and regular practice of physical activities to maintain the recommended glycemic control and prevent complications. Children and adolescents with T1D are more vulnerable to suboptimal self-management, failing to achieve the recommended glycemic control and showing a greater propensity to unbalanced emotional states [[3], [4]].

According to Boucher et al. [5], only 17% of people aged <18 years meet the glycated hemoglobin standards (HbA1c). On the other hand, recent studies have described that technological advances can help treat [6].

Chronic diseases directly affect individuals’ quality of life (QoL) as they impose several limitations. The health-related quality of life (HRQoL) evaluates individuals’ perception of their health and represents how severely diseases impact the physical, functional, emotional, and social aspects [[6], [7], [8], [9]]. According to Glocker et al. [10], individuals living with T1D who have suboptimal glycemic control showed reduced HRQoL.

Ensuring a good QoL for these individuals and avoiding complications requires frequent follow-ups with healthcare providers. Although education and support for diabetes self-management are essential for treatment, the availability and accessibility of this service differ between health systems [11].

In this context, digital interventions to manage T1D can help solve this challenge, and remote care enables frequent communication between patients and multiprofessional teams and data sharing, enabling more frequent adjustments in treatment [11].

Studies have shown that telemedicine in patients living with T1D offers a more agile and frequent contact with medical teams, increasing this population’s adherence to recommendations and improving disease management, especially in young adults [12].

Considering the growing influence of telemedicine in treating T1D, this research aims to evaluate the QoL and glycemic control of children and adolescents living with T1D before and after an intervention by a multiprofessional educational remote program.

## Methods

This prospective, interventional, and descriptive study was carried out at the Pediatric Endocrinology Service at the Hospital de Clínicas Complex at Universidade Federal do Paraná.

### *Participants*

Children and adolescents aged from 1 to 18 years who had been diagnosed with T1D for at least six months prior to the beginning of this study. Patients in the "honeymoon" phase, living in regions without internet coverage, or who were using a continuous insulin infusion system or a continuous interstitial glucose monitor (CGM), were excluded from this sample to assess the impact of this therapy on patients without access to these technologies. The sample was selected based on the weekly demand for routine outpatient care at the service, until a total of 50 individuals were included, based on the source population of approximately 150 children and adolescents with T1D who met the inclusion criteria.

Participants were chosen by convenience during routine medical consultations and individually randomized by lottery to compose the intervention group (IG) and the control group (CG). CG participants received standard outpatient follow-up, whereas IG participants underwent follow-ups via digital platform with a multidisciplinary team (Supplementary material – complete description of multidisciplinary services provided through a digital platform – Attachment 1) in addition to the standard follow-up over 180 days.

### *Instruments and data collection*

Socioeconomic, clinical, anthropometric, laboratory, physical activity level, psychological, and quality of life data were collected from the participants.

In total, three in-person evaluations were carried out at time 0 (T0), time 90 (T90), and time 180 (T180). In-person multiprofessional consultations, blood collection, and questionnaires were applied in the first and last of these. At T90, all participants had a CGM installed.

The PedsQL 3.0 Pediatric Quality of Life Questionnaire – Diabetes Mellitus Module was used to analyze participants’ QoL. It contains questions geared at children’s/adolescents’ QoL self-assessment and questionnaires to assess their QoL from the point of view of their parents/caregivers.

In addition to the QoL questionnaire, the Screen for Child Anxiety Emotional Related Disorders, the Bouchard activity diary, and a questionnaire developed by the research team were applied, covering socioeconomic data, including family composition, education level, housing, family income, government social benefits, and information on personal access to technology and the internet.

Data from DataSUS were used for economic classification, in which a low-income range was considered as families with an average per capita household income below half a minimum wage (Ministério da Saúde) [13].

Nutritional status was classified by BMI z-scores following the WHO classification (2007), whereas glycemic control was assessed following the 2022 ISPAD recommendations (HbA1c < 7%), and time in the therapeutic range following the Battelino et al.'s international consensus: > 70% (70-180 mg/dl); < 4% (< 70 lt; 70 mg/dl); < 1% (< 54 lt; 54 mg/dl); <25% (> 180 gt; 180 mg/dl), and < 5% (> 250 gt; 250 mg/dl) [[14], [15], [16]].

### *Ethical considerations*

The digital platform in this study consisted of an application (DOC®), that could be accessed by a computer, tablet, or smartphone 24 hours a day, only requiring internet network coverage. The digital platform was housed in a secure environment, meeting the requirements of Security Assurance Level 2 and Padrão ICP-Brasil.

The study was approved by the Human Research Ethics Committee at CHC-UFPR (CAAE No. 39133120.0.0000.0096). After agreeing to participate, parents or legal guardians signed the Informed Consent Form, and participants aged seven years or older signed the assent form.

### *Statistic*

Data were collected and recorded in an electronic spreadsheet, and were checked and exported to R, version 4.3.1. Children and adolescents were first classified into three groups: tertile 1: participants with the worst baseline perceptions in a particular QoL component, tertile 2: participants with intermediate perceptions, and tertile 3: those with the best perceptions. The effect of the intervention on QoL perception was evaluated by a linear regression in which the effects of the following factors were adjusted: age, time since diagnosis, gender, puberty, and presence of comorbidities.

The Student’s *t*-test was used to compare QoL and the other variables at T0 and T180 for paired samples, evaluating unpaired samples for individuals at both moments. A logistic regression was also applied to find associated variables (gender, pubertal stage, time since diagnosis, presence of comorbidities) with the outcome (change in QoL in the CG and IG at both times). Robust standard errors were calculated to circumvent possible problems in model specification. The Wald test was used to evaluate possible associations. Conclusions are based on a 5% significance level.

## Results

A total of 46 children and adolescents participated in this study, 52.2% of whom were boys with a mean age of 11.9 (± 2.1) years and a mean time since diagnosis of 4.5 (± 2.5) years. Most of the sample was at the pubertal phase at T0 and T180 (T0: 67.4% and T180: 82.6%). In addition to T1D, 43.5% also had some other comorbidity, the most common being dyslipidemia (35% of cases). Moreover, 15 participants belonged to the low-income socioeconomic group.

The IG consisted of 24 participants and the CG of 22. Boys comprised 58.3 and 45.45% of the IG and the CG, respectively. Table 1 describes participants’ clinical characteristics by comparing the groups at T0 and T180. Both groups had homogeneous general characteristics at T0, with no significant differences between them. Mean HbA1c exceeded the recommended level in both groups. The IG showed a significant increase in adherence to the CHCM after the educational intervention (p < 0.001), but the other evaluated parameters showed no significant changes at the end of the 180-day follow-up period.

**Table 1:** Clinical characteristics.

Characteristic	**CG**^a^**T0 Mean ± SD n (%)**	**CG**^a^**T180 Mean ± SD n (%)**	p	**IG**^b^**T0 Mean ± SD n (%)**	IG ^b^ T180 Mean ± SD n (%)	p
HbA1c	9.4±1.6	9.6±1.3	0.342	8.9±1.7	9.4±1.8	0.576
Eutrophic (BMI/age)	11 (50%)	10 (45.5%)	0.854	13(54.2%)	14(58.3%)	0.654
Carbohydrate Counting	11 (50%)	11 (50%)	1.000	10(41.7%)	14(58.3%)	<0.001
Level of Physical Activity	11 (50%)	10 (45.5%)	0.576	9(37.5%)	11(45.8%)	0.162
Presence of Anxiety	7 (31.8%)	5 (22.7%)	0.162	8 (33.3%)	5(20.8%)	0.083

Note: Student’s t-test for paired samples.

^a^Control Group (CG).

^b^Intervention Group (IG).

Table 2 shows the QoL of CG and IG participants and their guardians according to symptoms, barriers, and adherence to treatment, worries, and communication problems at T0 and T180 (showing no significant differences).

**Table 2:** Level of quality of life of participants and their parents and guardians at T0 and T180.

Characteristic	**CG**^a^**T0 Mean ± SD**	**CG**^a^**T180 Mean ± SD**	p	**IG**^b^**T0 Mean ± SD**	**IG**^b^**T180 Mean ± SD**	p
Diabetes Symptoms (Participants)	53.4±15.4	49.8±15.7	0.357	57±14.7	54.8±17.8	0.518
Barriers to Treatment (Participants)	54.8±21.8	51.4±25	0.397	55.2±18.6	55.6±18.6	0.909
Adherence to Treatment (Participants)	74.5±20.3	71.1±20.6	0.367	72.1±15.6	71.9±16.8	0.949
Worries (Participants)	39.6±30.1	33.2±20.7	0.277	41.5±28.8	37.1±20.9	0.544
Problems with Communication (Participants)	55±36.4	52.8±35.1	0.747	61.7±28.1	65.4±24.8	0.437
Total Quality of Life (Participants)	55.5±18.4	51.7±16.7	0.317	57.5±17.1	57±13.2	0.894
Diabetes Symptoms (Guardians)	51±16.2	51.7±15.9	0.811	51.7±17.2	52.5±15.4	0.844
Barriers to Treatment (Guardians)	42.5±15.5	45.3±21	0.524	43.6±22	49.3±20.3	0.237
Diabetes Symptoms (Guardians)	51±16.2	51.7±15.9	0.811	51.7±17.2	52.5±15.4	0.844
Barriers to Treatment (Guardians)	42.5±15.5	45.3±21	0.524	43.6±22	49.3±20.3	0.237
Adherence to Treatment (Guardians)	57.2±18.3	66.3±19.9	0.038	63±17.1	69.1±16.6	0.137
Worries (Guardians)	26±22.1	27.9±23	0.706	32.9±25.8	36.8±18.7	0.540
Problems with Communication (Guardians)	53.3±31.9	62.5±36.8	0.106	50.5±34	60.2±30	0.192
Total Quality of Life (Guardians)	46±16.1	50.7±14.9	0.060	48.3±18	53.6±12.1	0.104

Note: Student’s t-test for paired samples.

^a^Control Group (CG).

^b^Intervention Group (IG).

^c^p values from the comparison between the domains of participants and guardians: CG (T0): Barriers to Treatment 0.018; Adherence to Treatment < 0.001; Total Quality of Life 0.034. IG (T10): Barriers to Treatment 0.008; Adherence to Treatment < 0.029; Total Quality of Life 0.028. CG + IG (T0): Barriers to Treatment and Adherence to Treatment <0.001; Total Quality of Life 0.002. CG + IG (T180): Barriers to Treatment 0.041.

The highest QoL score for participants in both groups and their parents/guardians occurred in adherence to treatment. A significant increase in the score in this domain (p = 0.038) occurred in the CG from T0 to T180 according to the perception of parents and guardians. The remaining groups and times showed no significant differences.

A relevant finding refers to participants’ parents/guardians having a lower QoL score than their children in practically all categories. Parents and guardians showed a statistically lower perception of general QoL (9.318 (3.618;15.018); p = 0.002) than their children at T0 in barriers (11.957 (5.783;18.131); p < 0.001) and adherence to treatment (13.012 (7.284;18.740); p < 0.001). On the other hand, T180 only shows a significant difference in barriers to treatment (p = 0.041). Assessing agreement between all parents’ and guardians’ perceived QoL after the intervention obtained no significant results.

Assessing QoL based on sample tertiles obtained no significant differences between theIG and CG in the total QoL scores.

**Figure 1:**
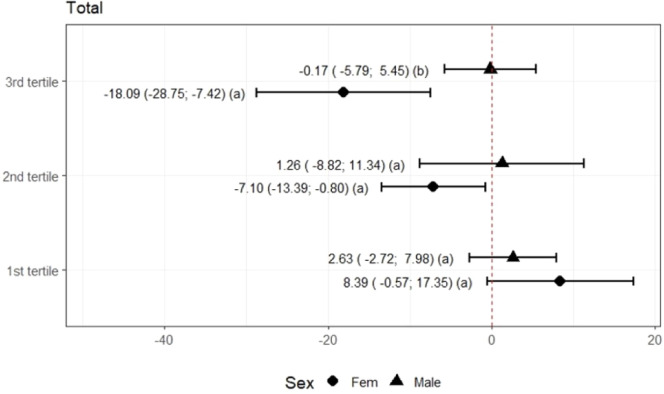
Comparison of total quality of life by tertiles between sexes. Note: Wald Test. ^a^ Tertile 1: participants with the worst perceptions of quality of life, tertile 2: intermediate perceptions, and tertile 3: best perceptions. Different letters indicate different average variations.

However, only analyzing QoL at T0 by gender obtained a lower HRQoL for girls in the third tertile (Figure 1). It was impossible to perform the analysis according to pubertal stage due to the small sample size of prepubertal participants. Socioeconomic factors did not influence the results.

**Figure 2:**
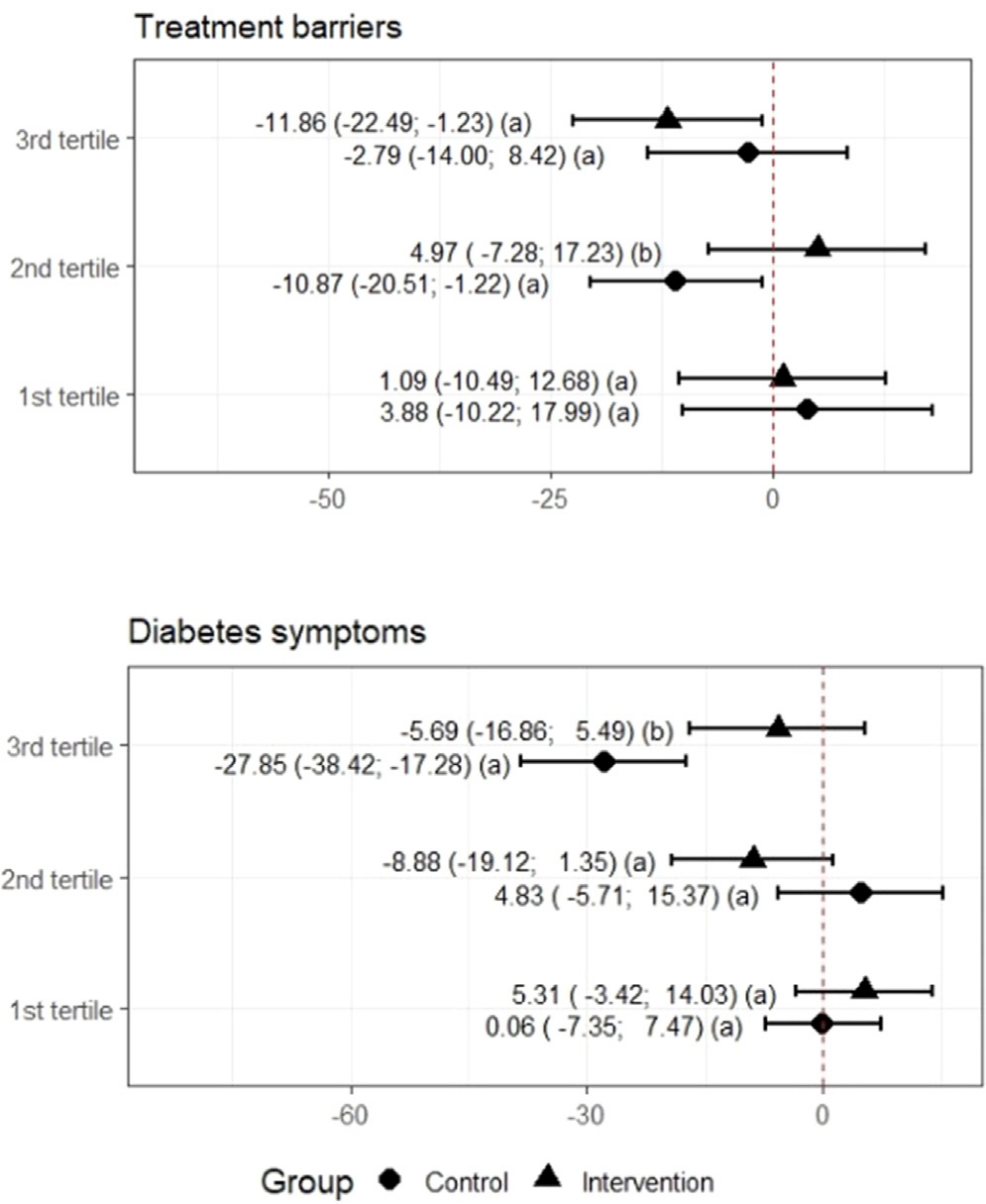
(A,B) Comparison of quality of life by tertiles between the intervention and control groups after the intervention regarding barriers to treatment and diabetes symptoms. Note: Wald Test. ^a^ Tertile 1: participants with the worst perceptions of quality of life, tertile 2: intermediate perceptions, and tertile 3: best perceptions. Different letters indicate different average variations. ^b^ Treatment Yes (Intervention) and Treatment No (Control Group).

The influence of the remote intervention at T180 on participants’ self-perceived QoL showed a difference between treatment for children and adolescents in the third tertile, more greatly reducing QoL associated with diabetes symptoms in CG (−27.55; 95%CI: −38.06; −17.65) than in IG (−5.69; 95%CI: −16.47; 5.10). Untreated adolescents also showed a greater reduction in QoL associated with barriers to treatment (−10.87; 95%CI: −20.18; −1.56) than their treated counterparts (4.97; 95%CI: −6.85; 16.80) in the second tertile (Figure 2). The adolescents in the other tertiles showed no significant differences between treatments.

Evaluating the effect of the intervention with a remote multi-professional educational program on participants’ glycemic control showed no significant variations in HbA1c between groups either at T0 or T180, and no significant correlations between HbA1c and QoL (p = 0.200).

After T90, this research could assess blood glucose by continuously monitoring these individuals, who showed a much lower time in the therapeutic range than recommended in both groups and times. The mean percentage of time in the therapeutic range of CGM use in the IG equaled 33.7 ± 14 in the first 30 days (T90-T120) and 34±13.8 in the last 30 days (T150-T180), whereas in the CG, such percentages totaled 28.7±10 and 27±7.6, respectively. A high percentage of time in severe hyperglycemia occurred, equaling 40.4±19.5 (T90-T120) and 40.3 ± 20.7 (T150-T180) in the IG and 46.4 ± 12.4 (T90-T120) and 48.8±11.9 (T150-T180) in the CG. Hypoglycemia duration and severe hypoglycemia remained within the recommended range. The percentage of time in the therapeutic range showed no significant difference between groups [CG (p = 0.187) and IG (p = 0.773)].

The results showed that, although the remote intervention did not directly impact QoL or glycemic control, it contributed to better adherence to CHCM, which may indirectly influence improved glycemic control. Moreover, it was observed that individuals who did not receive the intervention experienced a reduction in some QoL domains. Another relevant finding was a greater perceived burden among parents and guardians compared to the participants themselves, as well as among females, suggesting the need for educational strategies targeted at this group.

## Discussion

The total HRQoL scores in children and adolescents living with T1D and their parents and guardians are below those of other studies. A study carried out in Spain with 178 individuals living with T1D and a mean age of 10.6 years, found that they ranged from 64 to 79 in its several domains, that study differs from the present research as its mean HbA1c totaled 7.1%, i.e., most participants had better glycemic control than the ones in this sample, which can directly influence QoL [17].

Other studies on children and adolescents with suboptimal glycemic control found better HRQoL scores. Research in India with 107 individuals who had an average of 8.7% HbA1c, obtained total QoL scores equal to 68.2 in participants and to 64.4 in their parents/guardians [18]. Moreover, the scores in all domains exceeded 60.17. Another randomized trial carried out in New Zealand with 64 children and adolescents, HbA1c ≥ 9%, and in which the IG used a continuous glucose monitor showed a total QoL score above 60, total baseline QoL equaled 63.7 ± 15.6 and 68.9 ± 15.1 in their CG and IG, also above those in this research [5].

Khemakhem el al., 2020 [19]. evaluated the HRQoL of 48 individuals living with T1D, observing that girls and adolescents seem to have greater difficulty in managing the disease and worse QoL scores in all evaluated domains, a fact that corroborates the findings of this study since girls obtained lower HRQoL scores.

In a study carried out at the Joslin Diabetes Center with 169 adolescents with HbA1c 6.5-11.0%, female adolescents obtained significantly lower scores for female adolescents than in boys [20]. Data resembling those in a study carried out in Norway showed that lower HbA1c and the male gender were associated with better QoL in adolescents living with T1D [21].

This study found that the intervention by a multi-professional educational remote program failed to directly influence the increase in participants’ total HRQoL but found that individuals who received no intervention showed a significant reduction in HRQoL in some of the evaluated domains over time (especially for those who had a lower baseline HRQoL) in relation to IG.

A study conducted in Tunisia highlights that, according to the results of the PedsQL subscales, the most important difficulties for children and adolescents were related to barriers to treatment, followed by diabetes symptoms [19]. This research observed that its intervention significantly improved these dimensions.

The systematic review by Kavookjian J, et al. [9], stressed the importance of valuing the QoL questionnaire category results in addition to total scores since including subscale reports further assesses the impacts of diabetes education and self-management support in more specific elements.

This study observed a significant increase in adherence to the CCHCM, corroborating Boucher et al. [5], in that a multidisciplinary intervention brings long-term benefits and can thus optimize glycemic control and improve QoL. A 2023 meta-analysis that evaluated the efficacy of CHCM regarding the reduction of HbA1c in children living with T1D showed a significant reduction (p < 0.001) in HbA1c in the CHCM group in relation to the CG [22].

A 2020 meta-analysis concluded that mobile health interventions had a small effect on improving HbA1c, benefiting T1D patients by improving their QoL and concern about the disease. Moreover, participants in telemedicine care showed greater adherence to recommended care [12].

Another significant fact in this study that has been described in previous research refers to parents’ and guardians’ worse perception of their children’s QoL. These findings resembled those from Kuwait research with 455 children and adolescents with T1D, in which the parents had worse HRQoL than their children (79.06 ± 15.19 vs. 73.79 ± 15.17, p < 0.01) [3]. Studies in Italy and Norway have also shown that parents of children living with T1D perceived a lower total QoL than their children. Bratke et al. [21], concluded that differences in scores between children and parents suggest that parents may underestimate their children’s HRQoL.

Finally, it is important to highlight that in addition to the intervention of a multiprofessional educational remote program, all research participants used CGM during the last three months of this research. However, this failed to significantly influence the QoL of the evaluated individuals, as the time in the therapeutic range was much lower than recommended in both groups. A study carried out in New Zealand with a six-month intervention in the use of CGM also found no differences in glycemic control and total QoL scores after six months between its groups [5].

A randomized clinical trial, carried out in the United States with 143 children living with T1D, evaluated the effects of combining CGM with a family behavioral intervention versus CGM use alone on glycemic impact for six months, observing that the percentage of time in the therapeutic range between the groups showed no significant changes. Moreover, the percentage of time in the therapeutic range is thus also failing to meet recommendations [23].

A 2024 systematic found that telemedicine reduced HbA1c levels by 0.22 (p < 0.001) but failed to convincingly affect QoL scores. Other relevant results included a greater effect of telemedicine on HbA1c levels in studies involving children (p < 0.001), those that spanned more than six months (p < 0.001), those that used smartphone applications to communicate with participants (p < 0.001), and those that adjusted insulin doses via telemedicine (p < 0.001) [24].

These findings suggest that the treatment of diabetes far exceeds remote education and CGM use. Individuals with T1D, especially those with suboptimal glycemic control, face a considerable burden related to the disease, and improving their psychosocial outcomes may require a more long-term, accessible and individualized education.

In a study carried out in Congo to assess QoL and find risk factors associated with these individuals’ psychosocial experience, a lower QoL was found in participants with low socioeconomic status (p = 0.01) and in those with suboptimal glycemic control (p = 0.03) [25].

It is important to note that the participants of this research may have faced other socioeconomic and educational challenges in addition to T1D management. This study found that participants faced difficulties in managing technology.

When analyzing the influence of a multiprofessional educational remote program on the QoL of children and adolescents with T1D, the authors found no significant differences between groups. However, stratifying the data by sample tertiles showed a difference in those individuals in the CG with a better initial QoL and a worsening in diabetes symptoms in relation to the IG.

The results of this study also highlight the importance of considering gender as a relevant factor in assessing HRQoL in individuals living with T1D, as girls had significantly lower HRQoL scores.

Participants’ and parents’/guardians’ perceptions of HRQoL also differed, as caregivers showed lower scores in practically all categories. This finding highlights the impact of T1D on family dynamics and the need for multidisciplinary follow-up of both participants and their families.

Regarding the impact of the intervention on glycemic control, this research observed no improvements.

Although the educational intervention failed to improve participants’ general QoL, it significantly increased adherence to the CHCM in the IG, a factor that may contribute to improving glycemic control in individuals with T1D and influence their QoL in the long term.

In summary, the results of this study highlight the need to intensify strategies in diabetes management considering multifactorial aspects, such as socioeconomic aspects, educational level, family support, psychological characteristics, and access to technologies, among others, as several aspects can influence the search for optimal glycemic control and thus improve the HRQoL of these individuals.

## Funding sources

The present study was financed by Blue Mountain Participações Ltda., with funding for equipment purchase. Two of the authors received a 3-year scholarship for a PhD program from the Coordination for the Improvement of Higher Education Personnel (CAPES). The founders had no role in the design, data collection, data analysis, and reporting of this study.

## Data availability statement

The data that support the findings of this study are available from the corresponding author.

## Conflicts of interest

The authors declare no conflicts of interest.

## Appendix Supplementary materials

Image, application 1
