# Supplementary material for: Evaluation of the quality of life of children and adolescents with type 1 diabetes mellitus before and after an intervention with a remote multiprofessional educational program
Source: J Pediatr (Rio J). 2026 Apr 3;102(3):101537. doi: 10.1016/j.jped.2026.101537 (PMC13089161; doi:10.1016/j.jped.2026.101537)
Supplement: Supplementary file 1 [file mmc1.docx]

**JPED-D-25-00363_ SUPPLEMENTARY MATERIAL**

| **SUPPLEMENTARY MATERIAL – COMPLETE DESCRIPTION OF THE MULTIDISCIPLINARY APPOINTMENTS CONDUCTED THROUGH A DIGITAL PLATFORM**   \| COMPOSITION OF THE TEAM AND FOLLOW-UP PLAN FOR MULTIDISCIPLINARY CONSULTATIONS VIA APPLICATION \| \| \| \| \| --- \| --- \| --- \| --- \| \| **Multidisciplinary Team Professional** \| **Number of Teleconsultations** \| **Duration of Teleconsultations** \| **Teleconsultation Approach** \| \| **Doctor** \| 4 \| 30 minutes \| Topics addressed included the patient’s overall health, inter-appointment intercurrent events, frequency of hypoglycemia or hyperglycemia, diet, physical activity, school performance, insulin doses in use, and blood glucose monitoring. \| \| **Nutritionist** \| 10 \| 30 minutes \| Topics addressed included guidance on care during hypoglycemia and hyperglycemia, carbohydrate, protein, and fat counting, diet and physical activity, eating at school, eating at parties, and eating while traveling. \| \| **Nurse/Diabetes Educator** \| 4 \| 30 minutes \| Topics addressed included the use and storage of insulin, injection techniques and rotation, and clarification of questions. \| \| **Psychologist** \| 5 \| 30 minutes \| Topics addressed included recognizing emotions and practical emotional regulation strategies adapted to the age group. The activities were designed in a playful manner, using visual resources, metaphors, and age-appropriate language for the children. \| \| **Physical educator** \| 4 \| 30 minutes \| Topics addressed included guidance and assessment of adherence to physical activity practices, glycemic targets before and during physical activity, and prevention of hypoglycemia and hydration during physical activity. \| |
| --- | --- | --- | --- | --- | --- | --- | --- | --- | --- | --- | --- | --- | --- | --- | --- | --- | --- | --- | --- | --- | --- | --- | --- | --- | --- | --- | --- | --- |
